# Editors’ Choice 2023

**DOI:** 10.1038/s44172-023-00151-7

**Published:** 2023-12-21

**Authors:** Miranda Vinay, Liwen Sang, Jianhua (Joshua) Tong, Or Perlman, Rosamund Daw, Carmine Galasso, Mengying Su, Damien Querlioz, Liangfei Tian, Anastasiia Vasylchenkova, Yu-Cheng Chen, Chaoran Huang

**Affiliations:** 1 Communications Engineering, Springer Nature, Berlin, Germany; 2 International Center for Materials Nanoarchitectonics, National Institute for Materials Science (NIMS), Tsukuba, Japan; 3 Department of Materials Science and Engineering, Clemson University, Clemson, SC USA; 4 Department of Biomedical Engineering and Sagol School of Neuroscience, Tel Aviv University, Tel Aviv, Israel; 5 Communications Engineering, Springer Nature, London, UK; 6 Department of Civil, Environmental & Geomatic Engineering, University College London (UCL), London, UK; 7 Communications Engineering, Springer Nature, Shanghai, China; 8 Université Paris-Saclay, CNRS, Centre de Nanosciences et de Nanotechnologies, Palaiseau, France; 9 Department of Biomedical Engineering, Zhejiang University, Hangzhou, China; 10 School of Electrical and Electronics Engineering, Nanyang Technological University, Singapore, Singapore; 11 Department of Electronic Engineering, the Chinese University of Hong Kong, Hong Kong SAR, China

**Keywords:** Engineering

## Abstract

The Editorial Board and Editorial Team are delighted to present a selection of short Research Highlights describing some of our favourite *Communications Engineering* publications of 2023.

As 2023 draws to a close, we highlight some of the great stories of engineering insight and solutions that we have published over the year. The final selection covers topics from across the engineering spectrum capturing our multidisciplinary scope. They are presented in order of publication date from earliest to latest in the year. And if you want even more, head over to our *Nature Portfolio* collection. There you will find coverage by the *Nature* journals and *Nature*’s multimedia team of other exciting *Communications Engineering* content: https://www.nature.com/collections/commsengcoverage.

## Electrically stimulated optical spectroscopy of interface defects in wide-bandgap field-effect transistors; Maximilian Feil et al.

Wide-bandgap semiconductor devices, and particularly their transistors, are key components of compact and efficient high-voltage electronics. They can be attractive alternatives to silicon-based electronics due to their higher breakdown field^1^. However, these devices are often composed of several layers. Wide-bandgap materials tend to form many defects at the interface between the semiconductor and the insulator—sometimes at 100 times the defect density of the silicon/silicon dioxide interface!^2^—lowering their parameter stability. Therefore detecting these interfacial defects as they evolve over time is an important characterisation for fabricated devices.

**Fig. 1:**
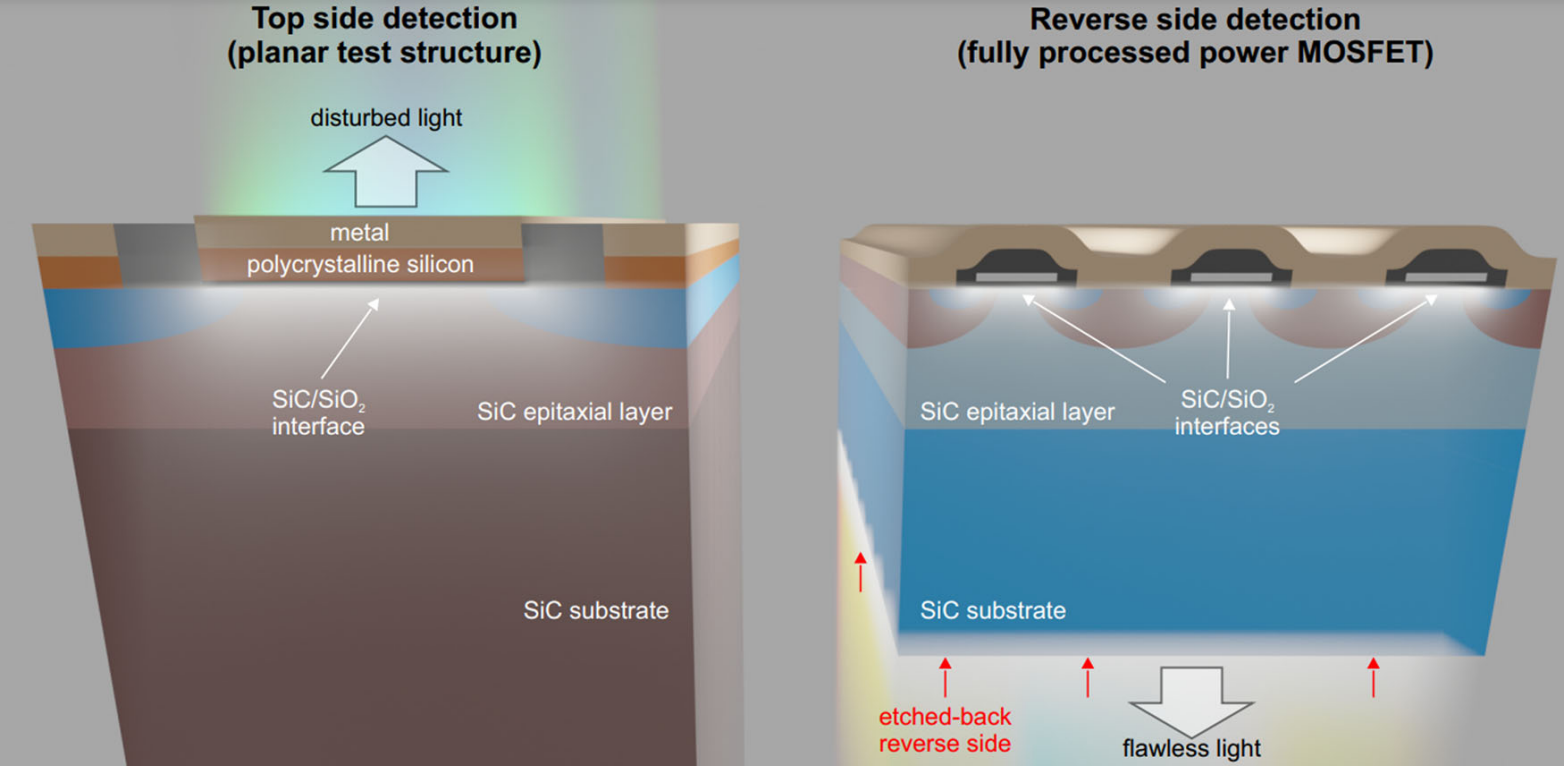
Illustration of optical top-side detection (approach reported in previous studies) and reverse-side detection (approach reported here). Reused from^3^.

Measuring point defects at the interface after fabrication often involve destroying the delicate device architecture or fabricating dedicated test structures to investigate the interface. An article by Feil et al.^3^ published a clever alternative in *Communications Engineering*, etching back the device substrate to spectroscopically probe the field-effect stimulated radiative defect transitions from the underside of wide-bandgap semiconductor metal-oxide-semiconductor field-effect transistors. A contrast between their approaches and those of previous works can be found in Fig. 1.

Their method, tested on commercially available 4H-SiC MOSFETs, was etched first with nitric acid and then with aqua regia to remove the copper frame and underlying substrate, and finished by polishing with diamond paste. This removed the drain contact of the device. Then, using a custom-built measurement system and emission spectroscopy, they were able to measure the linearity of photon emission with increasing switching frequency. They discovered a one-to-one correlation between photon emission and threshold voltage shift, which can even be measured during device operation. They further find that the resulting emission spectrum can give insight into the energetic position of the involved defects within the semiconductor’s bandgap. Their work brings device characterisation one step closer to all-optical in situ methods.

In the future, analysing emitted photons from the semiconductor-insulator interface will give a greater window into the behaviour and consequences of defects in wide-bandgap semiconductor devices. This makes mitigating or exploiting those defects for their effects on device performance easier, bringing us closer to realising their potential in long range electric vehicles and renewable energy generation. **Miranda Vinay**

## Vertical GeSn nanowire MOSFETs for CMOS beyond silicon; Mingshan Liu et al.

As modern transistors continue to scale down in size, conventional complementary metal oxide semiconductor (CMOS) technology on Si is reaching its physical limits. Alternative technologies are increasingly becoming research interests to extend Moore’s law.^4,5^ In the CMOS configuration, both n-channel and p-channel MOS field effect transistors (MOSFETs) with high mobilities are promising for high performance with low power consumption. Another requirement is the capability of integration with the current Si technology. However, it is difficult for one semiconductor system to satisfy all the above requirements.

This year, Liu et al. have reported^6^ in *Communications Engineering*, the development of high-performance MOSFETs based on GeSn semiconductor alloys grown on Si. They achieved the operation of both p-channel and n-channel behaviour with a vertical nanowire gate-all-around FET structure. The authors engineered the transistor structure for GeSn/Ge heterojunctions and fabricated nanowire FETs. The p-FET performance is improved by exploiting a small band gap of GeSn as a source yielding high injection velocities. The electron mobility of the n-FETs is improved by using GeSn as channel material.

They present for the first time a CMOS inverter based on GeSn nanowire transistors, demonstrating the integration potential and logic gates applications. Especially, at cryogenic temperatures, the developed full GeSn FET shows a much better performance than the current Si nanowire MOSFETs.

This is the pioneer report on GeSn nanowire CMOS technology. The demonstration of a GeSn inverter and steep switching at cryogenic temperatures shows the high potential of this material system for the future beyond Si CMOS logic and quantum computing applications. **Liwen Sang**

## Heavy metal removal from coal fly ash for low carbon footprint cement; Bing Deng et al.

Building materials are the third-largest source of anthropogenic carbon dioxide (CO2). The global CO2 emission during cement production represents ~8% of the total GHG emissions. Therefore, finding alternative materials with a lower carbon footprint to replace or partially substitute ordinary Portland cement (OPC) (the most extensively used building material) has been renewed recently^7^. Because of the widespread availability and ultralow cost, coal fly ash (CFA) is considered an attractive diluent additive in OPC^8^. However, CFA usually contains heavy metals, such as cadmium (Cd), cobalt (Co), copper (Cu), nickel (Ni), lead (Pb), and mercury (Hg), which may classify CFAs as hazardous waste and engender environmental concerns. Current methods for heavy metal removal mostly rely on the acid washing processes, suffering from the consumption of chemicals and generation of large volumes of wastewater.

To address this problem, Deng et al. reported in *Communications Engineering*^9^ a rapid and water-free process for heavy metal removal from CFAs for low-carbon footprint cement. Flash Joule Heating (FJH), an efficient high-temperature technology^10^, was used. The FJH process used an electric pulse to ramp up the temperature to around 3000 °C within one second, enabling the evaporative removal of heavy metals with efficiencies of 70–90% for As, Cd, Co, Cu, and Pb. The purified CFA is partially substituted in Portland cement, showing enhanced strength and less heavy metal leakage under acid leaching. Techno-economic analysis showed that the process was energy-efficient, costing around $21 per ton of electrical energy. Life cycle analysis revealed that using CFA in cement reduced GHG emissions by around 30% and heavy metal emissions by around 41%, compared to the current waste management practices (landfilling).

Importantly, the FJH strategy is also applicable for decontaminating other wastes like bauxite residue. The ongoing commercial scale-up of the FJH process makes it appealing for the decontamination and valorisation of large-scale industrial wastes. **Jianhua (Joshua) Tong**

## GaNDLF: the generally nuanced deep learning framework for scalable end-to-end clinical workflows; Sarthak Pati et al.

Deep learning (DL) is gradually positioning itself as a powerful tool for making new scientific discoveries and accelerating research. However, for scientists, physicians, and engineers outside the computational research community, the road to successfully applying DL strategies for their core research questions may prove bumpy, time-consuming, and sometimes impractical. According to a recent *Nature* survey^11^ on AI in science, with more than 1600 scientists responding, one of the main barriers to including AI in science is “the lack of skills or skilled researchers”.

This year, as part of an international multi-institution industry-academic joint effort, Pati et al. have reported^12^ in *Communications Engineering*, a community-driven open-source generally nuanced deep learning framework (GaNDLF), which drastically simplifies the development of DL models, using a “zero/low-code” approach. Focused on healthcare applications, GaNDLF provides a one-stop-shop for DL model development, training, inference and deployment without requiring extensive technical background. The framework orchestrates various previously established toolkits and libraries under its hood while dismantling the expertise needed to operate each library. The paper presented various clinical applications involving CT, MRI, and histological data, targeting a variety of AI workloads or tasks, including lesion/organ segmentation, tumour type classification, and brain age regression.

The framework contains built-in modules for handling class imbalance, training data augmentation, and specialised pre/post-processing steps, which allow users to focus on answering research questions at scale using well-established machine learning practices. When implementing DL in science, a significant concern is the “black-box” nature of the AI model. In that context, one of the main appealing aspects of GaNDLF is its integrated interpretability tool that generates attention maps, which visualise the region in the input data that primarily affected the neural network prediction.

Importantly, while GaNDLF makes research and development more accessible and easier to the broad audience, it is important that researchers are familiar with data science concepts, which are critical to prevent misuse and ensure reproducibility. However, once a basic understanding of DL is obtained, a framework like GaNDLF can enable a broader audience to join and benefit from the application of AI in research without the requirement to code every part of a DL pipeline. **Or Perlman**

## Lab2Field transfer of a robotic raspberry harvester enabled by a soft sensorized physical twin; Kai Junge et al.

**Fig. 2:**
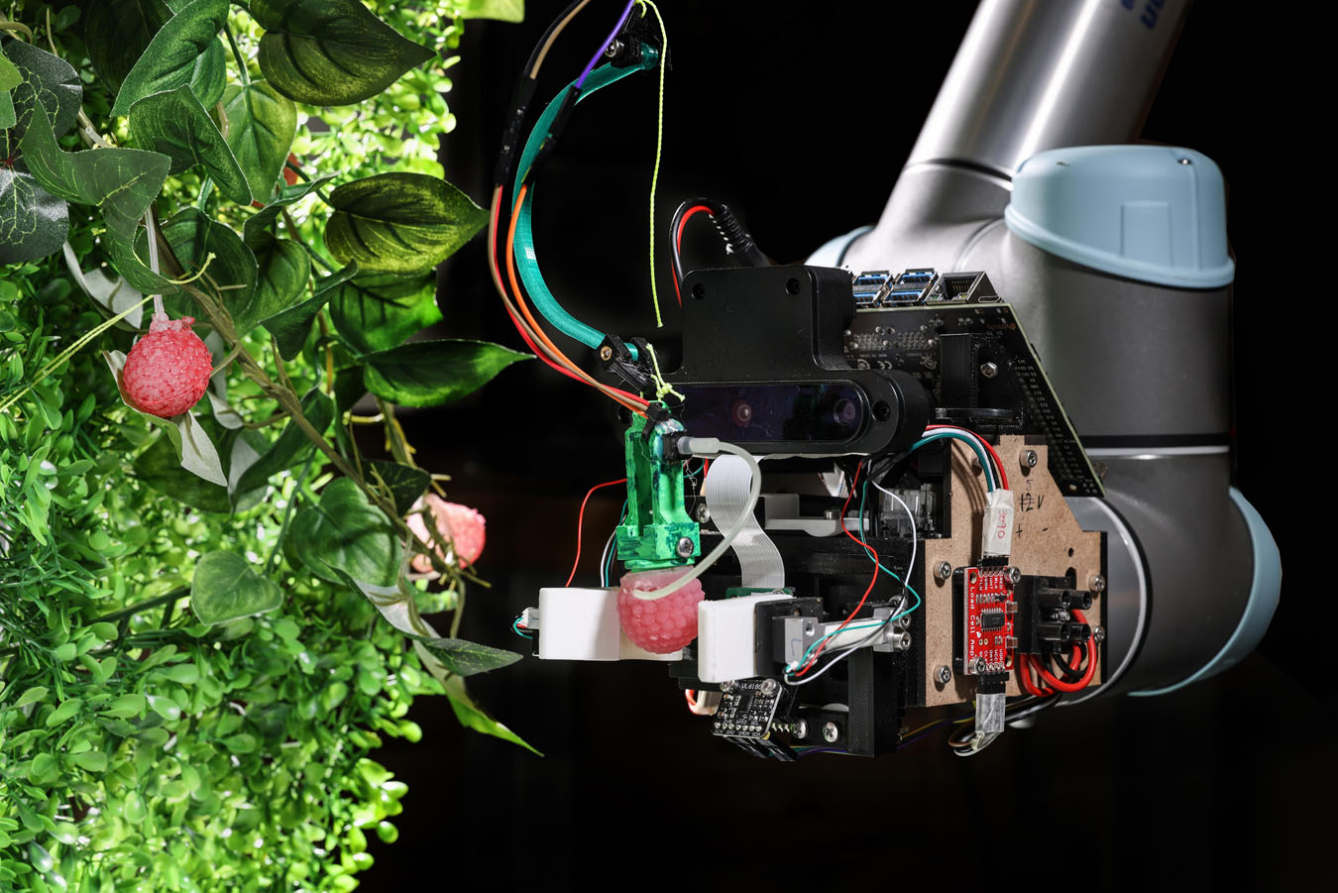
Photograph of the physical twin of Raspberry and robot in training (credit: Kai Junge, Alain Herzog).

Robots could help improve the efficiency of fruit picking, making the most of every harvest. However, selecting ripe fruit and picking without damaging the product is a challenging task for a robot. Kai Junge and colleagues reported in *Communications Engineering*^13^ an artificial plant complete with fake raspberries designed to allow robots to be trained to pick fruit throughout the year, and not just during summer when ripe fruit is available for field trials (see Fig. 2).

The soft physical twin of the fruit, fabricated from silicone, has integrated fluidic sensors, enabling the detection and subsequent recording of the forces applied to the fruit when a human picks it from the plant. A magnet is placed in both the fruit and the plant, the separation distance of which can be tuned via a screw, to vary the maximum force required to pull the fruit from the plant. Three different pulling forces were set to reflect different degrees of ripeness of the fruit.

The sensory information acquired during the human action of picking the fake fruit from its plant is then fed to a controller with the goal of re-creating the same forces in the subsequent robot-picking action. The compression force when gripping the fruit is set as the minimum required to pull the fruit without squashing it. Meanwhile, the controller is tuned to apply the appropriate pulling force such that only ripe fruit is harvested. Unripe fruit which require greater forces to detach from the plant will remain attached. The physical twin can also be used to help train the visual detection and motion planning of the robot towards the fruit.

In initial tests, the harvesting controller with no further tuning or adjustments was tested on 25 raspberries with a range of size and ripeness: 80% were successfully picked with no or minimal damage. When testing both the raspberry detection and harvesting performance of the robot without human intervention, the robot successfully picked four raspberries within seven attempts.

The paper is a creative combination of soft robotics and biomimetics, with potential practical benefits in efficient robotic training. It contributes ideas toward the ultimate goal of a more productive, cost-effective fruit harvest. **Rosamund Daw**

## The silent impact of underground climate change on civil infrastructure; Alessandro F. Rotta Loria

A study in *Communications Engineering* this year^14^ explored the impact of underground climate change, specifically subsurface heat islands, on civil infrastructure. Subsurface heat islands refer to areas of higher temperatures that occur underground, particularly in urban environments. For instance, buildings and infrastructure continually disperse heat into the ground, a byproduct of thermal losses linked with indoor heating and the operation of appliances. This form of “underground climate change” can significantly impact public health (e.g., thermal discomfort and heat-induced diseases) and transportation systems (e.g., overheated subway rails forcing trains to slow down or stop to avoid incidents with significant economic losses associated with delays).

The research centred around the Chicago Illionis’ Loop district and employed advanced 3D computer modelling and temperature sensors (i.e., a *digital twin* of the case-study area^15^) to examine the ground temperature variations, deformations and displacements caused by subsurface heat islands. The findings reveal deformations and displacements that potentially may conflict with and jeopardise the operational requirements of civil structures and infrastructure systems, depending on the specific case.

Without adequately considering these underground climate changes, buildings and infrastructures in densely populated areas could be at risk of structural damage and disruptions. Nevertheless, the study also suggests that subsurface heat islands present an opportunity to harness or minimise waste heat in the ground. By implementing innovative geothermal technologies, cities can repurpose this excess heat for various aims, including heating buildings and supplying heat to the district, contributing to decarbonisation and sustainability of urban areas.

Therefore, it is crucial to consider the impact of these underground climate changes in future urban planning and design strategies as well as in decision-making for infrastructure retrofit/adaptation interventions (e.g., thermal insulation of underground building enclosures) towards more resilient infrastructure^16^.

As Rotta Loria proposes, addressing these issues in a holistic and integrated manner, supported by engineering evidence, is essential for achieving sustainable development. I particularly appreciated this aspect of the study, together with its forward-looking and action-oriented approach to resilient infrastructure. This involves not just anticipating future changes but also leveraging the opportunities that arise from climate-related threats. This proactive approach promotes tactics that enhance resilience and sustainability at a broader societal level. **Carmine Galasso**

## The economic value of augmentative exoskeletons and their assistance; Roberto Leo Medrano et al.

Augmentative exoskeletons can assist human beings to surpass the limitations of human abilities. Lower-limb exoskeletons, for instance, can greatly enhance human mobility by providing mechanical support to leg joints while in harmony with the human neuromotor system.

The metrics for evaluating exoskeletons typically focus on objective and physical factors, such as the reduction of metabolic energy expenditure during locomotion. However, it is also crucial to consider the wearer’s perception, as it plays an important role regarding the successful integration and acceptance of exoskeletons in society.

R. Leo Medrano and colleagues introduced an unusual economic approach to evaluate the effectiveness of exoskeletons in a recent publication at *Communications Engineering*^17^. They utilised a Vickrey auction format to determine the value that each user places on the exoskeleton’s assistance. In other words, the participants were asked to consider how much they were willing to pay for the use of the assistive device. The study involved 16 able-bodied participants who walked on a treadmill with a 10˚ incline, with and without exoskeleton assistance. However, to be eligible for compensation, they had to complete a 2-min walking task. If they lost the auction, they were allowed to take a rest until the next auction. As participants grew tired, the value of the exoskeleton increased. The concept of marginal value, represented by the difference between the price-to-walk with and without assistance, can quantify the economic value of augmentative exoskeletons in an easy-to-understand unit of monetary currency.

Results show that the marginal value applied by the exoskeleton is $3.40/h, which, although positive, is relatively modest. This suggests that ankle exoskeletons may not offer significant advantages to individuals engaging in uphill walking over short periods. The assistance itself did appear valuable—having a value of $19.80/h—however, this value was offset by the cost of wearing the added weight. The results provide insights to exoskeletons designers to enhance the practical impact of their devices. For example, they can consider incorporating factors like inertia and comfort into their designs or focus on users who derive greater economic value from the exoskeletons. **Mengying Su**

## Weighted spin torque nano-oscillator system for neuromorphic computing; Tim Böhnert et al.

Spin electronics-based devices, which leverage both the charge and the magnetic moment of electrons, offer outstanding potential for neuromorphic computing. The intrinsic physics of these devices encompasses a wide range of behaviours—from memory retention (both long-term and short-term) to oscillation, resonance, wave propagation, and controlled stochasticity. These phenomena can emulate various types of artificial neurons and synapses^18^. However, the implementation of each of these different behaviours typically necessitates distinct devices, each crafted from a specially optimised, complex stack of magnetic and non-magnetic materials. This diversity poses challenges in integrating multiple spin electronics phenomena within a single electronic chip. A recent advancement in this domain is the radiofrequency spintronic neural network presented by Ross et al.^19^, which uses identical devices for both neurons and synapses. Yet, this approach requires external implementation of the synaptic memory feature.

In a groundbreaking development reported in *Communications Engineering*, Böhnert and colleagues have engineered a versatile stack^20^. It is optimised for vortex-based spin torque oscillators. In these devices, a naturally occurring magnetic “vortex” exhibits complex oscillations in response to input stimuli, implementing a sophisticated neuron behaviour. However, the research demonstrates that this same material stack, with modified lateral dimensions, can also implement magnetic memories, usable as artificial synapses. This dual functionality was achieved through a meticulous optimisation of the magnetic anisotropy within the structure, avoiding the vortex formation in the memory case. Moreover, the team successfully implemented a small-scale neural network using both types of devices. The next step, as proposed in this paper, involves integrating this technology into a CMOS platform and scaling up the neural network (in the paper, scaled-up neural networks are simulated and not implemented experimentally). This research marks a substantial stride towards integrating diverse neuromorphic concepts on-chip, paving the way for more sophisticated and integrated neuromorphic computing solutions. It also highlights the potential of neuromorphic spintronics. **Damien Querlioz**

## Electroacoustic tomography for real-time visualisation of electrical field dynamics in deep tissue during electroporation; Lifei Xu et al.

**Fig. 3:**
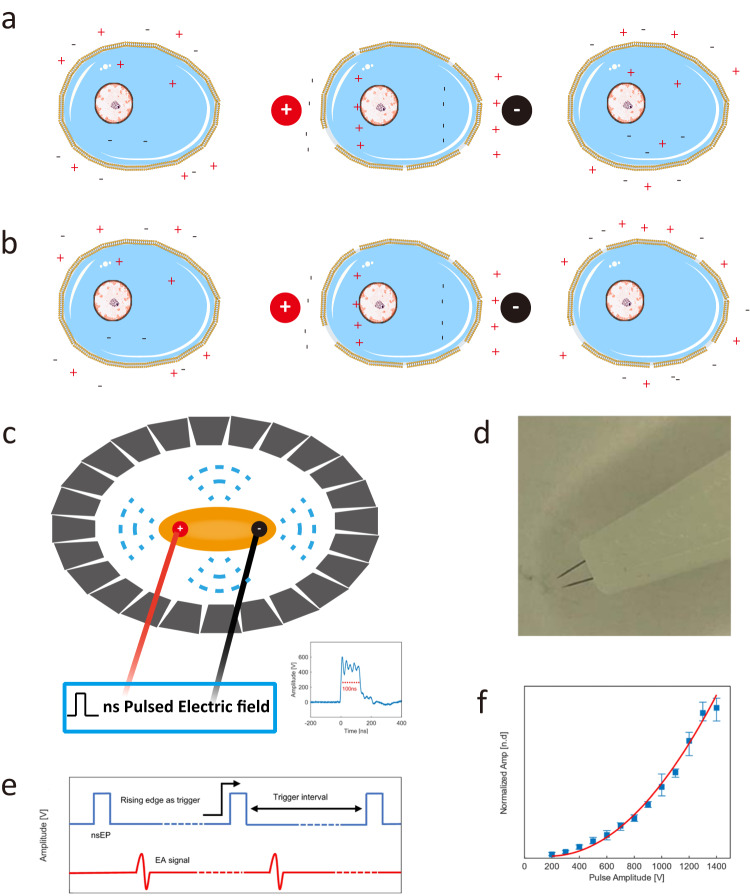
Electroporation and Electroacoustic tomography. **a**, **b** Schematic of reversible (**a**)/irreversible (**b**) electroporation. Red circles: positive electrodes, black circles: negative electrodes. **c** Schematic of tissue (yellow ellipse) generating an acoustic signal (blue dashed line) in response to an electric field, which is received by a ring transducer array (grey trapezoidal array). **d** Electrodes used in the electroacoustic tomography system with ~100 µm electrode diameter. **e** A periodic rectangular waveform with peak-to-peak voltages of 500 *V*_pp_ (blue), repeating at 1000 Hz for nsPEF electroporation generates an electroacoustic signal (red). **f** The relationship between electroacoustic signal strength and input voltage follows an exponential function, which is associated with electrical energy deposition and the electroporation effect. (Credit: Liangfei Tian, some panels re-used from ref. ^23^).

Electroporation is a technique that increases the permeability of the membrane of a target cell. The application of a pulsed electric field creates microscopic pores in the cell membrane, allowing exogenous substances such as protein molecules, nucleic acids, and drugs to enter the cell. Micropores induced by the electric field close after a period of time allowing the cell to remain active (Fig. 3a). Electroporation has a variety of potential biomedical applications. However, if the intensity of the pulsed electric field is too high, it will lead to irreversible membrane perforation and cell death (Fig. 3b). So monitoring the electric field during treatment is vital. However, this is challenging to achieve in real-time with current imaging methods^21,22^. In a publication in *Communications Engineering*^23^, Lifei Xu and colleagues explored the use of electroacoustic tomography in the form of nanosecond pulsed electric field excitation for broadband ultrasound detection, with the potential to monitor electric field distributions and electroporation in soft tissues at high resolution.

When short pulses of intense electrical energy are applied to biological tissue, some of the electrical energy is absorbed and converted to heat, resulting in a temperature rise that induces an acoustic signal due to the thermoelastic expansion of the tissue. To generate sufficiently short voltage pulses, Xu and colleagues designed a high-voltage nanosecond pulse generator to deliver electrical energy to the tissue (Fig. 3c). The diameter of the individual electrodes was around 100 micrometres, a key to improving the spatial resolution for real-time monitoring of electroporation (Fig. 3d). To receive the ultrasound signals, they fabricated an ultrasound transducer array with 128 units and a centre frequency of 5 MHz, capable of a pulse width of 100 ns and a repetition frequency of 1 MHz (Fig. 3e). The high repetition frequency allowed the system to achieve a high temporal resolution. As the amplitude of the electroacoustic signal is directly proportional to the input voltage, EAT is capable of monitoring electroporation (Fig. 3f). The platform achieves a tissue imaging depth of more than 7.5 cm, a spatial resolution lower than 0.2 mm, and a temporal resolution of 10 ms.

EAT has the potential to establish electric field strength thresholds for reversible electroporation for gene therapy and transdermal drug delivery to minimise unnecessary damage and reduce treatment risks. Furthermore, it can be applied in irreversible electroporation treatments, such as tumour ablation, to provide guidance on the spatial distribution of cellular apoptosis. Importantly, this approach holds promise for compatibility with existing ultrasound clinical imaging equipment, enhancing cost-effectiveness and convenience. **Liangfei Tian and Xingyu Jiang**

## Celestial compass sensor mimics the insect eye for navigation under cloudy and occluded skies, Evripidis Gkanias et al.

Solar light is the most powerful resource on earth, offering tremendous energy for all plants and living species to grow and sustain. It is also an important resource for insects and animals to navigate themselves in nature. For instance, bats use the way the sun’s light is scattered in the atmosphere at sunset to calibrate their internal magnetic compass, which helps them to fly in the right directions^24^. Like many other insects, honeybees can find their way because their eyes can find the position of the Sun in the sky even when it is hidden in the dense clouds^25^. How can these be achieved without the help of GPS or navigation electronics?

These can be achieved because their eyes are sensitive to light polarisation. There is a hypothesis that the Viking’s achievements in navigating the sea were enabled by a similar technique^26^. The famous sunstone, most likely Iceland spat, could be used to evaluate the degree of polarisation when looking through it at the sky. Finding the Sun’s location and knowing the time was enough to find the North. It could let the Vikings build their ship’s route and reach remote locations.

**Fig. 4:**
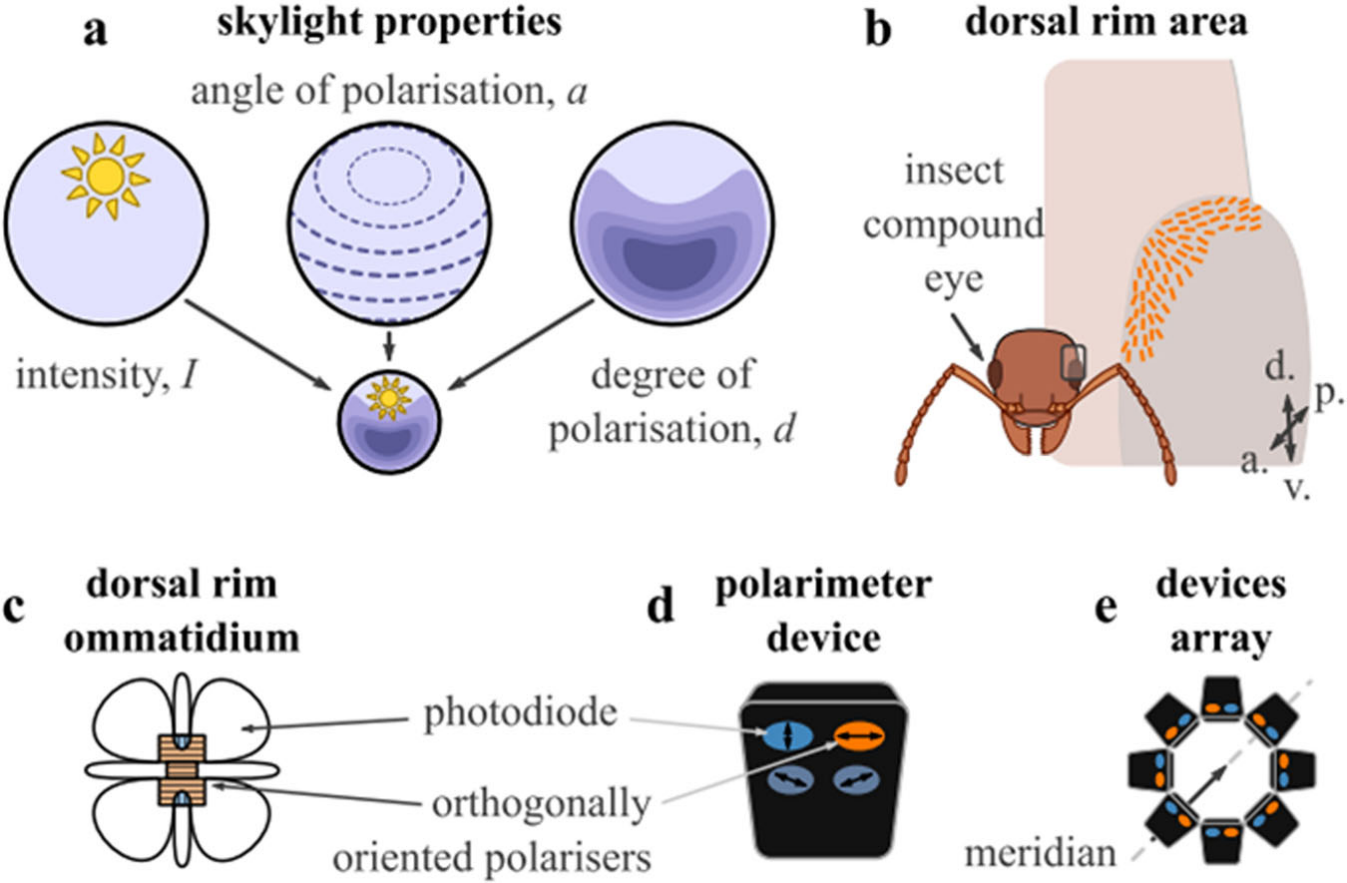
Overview of the design concept of the bio-inspired celestial compass sensor, re-used from ref. ^27^. **a** Skylight varies in light intensity (*I*), angle (*a*) and degree (*d*) of polarisation. **b** The compound eyes of insects filter light using a fan-like distribution of specialised ommatidia. These point in different directions in the sky and cover up to 120° field of view. **c** In each ommatidium, the skylight is filtered by orthogonally oriented microvilli and then captured by two groups of photoreceptor cells. **d** Our polarisation axis analyser mimics this function by filtering the skylight with two orthogonal polarizers before being captured by the photodiodes. **e** Our sensor has eight PAAs in a ring arrangement, elevated by 45°.

Almost like Vikings, inspired by the structure of insects’ eyes, a group of researchers from Edinburgh, led by Dr Gkanias, designed a celestial compass mimicking the insects’ eye composition^27^ (Fig. 4). A circle of tilted polarimeters with orthogonally directed polarisation axes was developed. Comparing the detected properties of light between the sensors, the accompanying algorithm finds the local maxima for the light intensity and for the degree of polarisation. The light intensity is maximal towards the Sun while the degree of polarisation is the highest opposite to the Sun. This additional feature resolves the ambiguity of the opposite direction along the solar meridian.

The sensor shows remarkable results, achieving high accuracy in detecting the north or in anchoring to the reference point in the clear sky, under the broken and dense clouds, in the densely built surroundings and under the trees. The presented celestial compass is built from off-the-shelf components and therefore low cost and easy to build. It avoids the usage of heavy magnets and is therefore lightweight. While the prototype is large to ease the customisation, it can be miniaturised on the base of printed circuit boards or complementary metal-oxide semiconductor platforms. The sensor provides a robust compass system that could potentially assist in future autonomous vehicles and devices. **Anastasiia Vasylchenkova and Yu-Cheng Chen**

## Photonic signal processor based on a Kerr microcomb for real-time video image processing; Mengxi Tan et al.

Real-time image and video processing is a foundational technology empowering diverse applications, such as machine vision, robotics, surveillance, augmented reality, and medical imaging. These applications demand immediate processing of extensive real-world information, placing significant demands on processing speed and throughput. Although electrical digital signal processors (DSP) are well-established, they encounter speed limitations attributed to the von Neumann bottleneck. In response, Menxi Tan and colleagues developed a photonic processor that enables real-time video image processing at an ultra-high speed of 17 Terabits/s, capable of concurrently processing almost 400,000 videos^28^. Notably, the system is not only high-speed but also highly reconfigurable and programmable. It can execute up to 34 functions in real-time without requiring any changes to the physical hardware. These include essential image processing functions like edge enhancement, edge detection, and motion blur correction.

The exceptional performances result from leveraging the massive parallelism and high bandwidth of lightwaves, which are unique properties inherent to light. The key enabling device is an integrated Kerr soliton crystal micro-comb, which produces 95 discrete taps or wavelengths, each supporting a data rate of 64 GigaBaud (pixels/s). The micro-comb, generated by a compact micro-scale resonator, has driven significant breakthroughs in diverse fields, including metrology, spectroscopy, telecommunications, quantum information processing, and light detection & ranging (LIDAR).

This research builds upon the authors’ earlier study published in *Nature*^29^ where they introduced a photonic convolutional neural network with a computing throughput of 11 Tera operations per second. This work not only represents a leap forward in fundamental photonic computing and signal processing but also translates into tangible and substantial benefits for industrial and real-world applications where real-time image and video processing is mission-critical. **Chaoran Huang**
